# Patient-reported dyspnea and health predict waitlist mortality in patients waiting for lung transplantation in Japan

**DOI:** 10.1186/s12931-021-01715-x

**Published:** 2021-04-21

**Authors:** Masaki Ikeda, Toru Oga, Toyofumi F. Chen-Yoshikawa, Junko Tokuno, Takahiro Oto, Tomoyo Okawa, Yoshinori Okada, Miki Akiba, Satona Tanaka, Yoshito Yamada, Yojiro Yutaka, Akihiro Ohsumi, Daisuke Nakajima, Masatsugu Hamaji, Maki Isomi, Kazuo Chin, Hiroshi Date

**Affiliations:** 1 Department of Thoracic Surgery, Graduate School of Medicine, Kyoto University, Kyoto, Japan; 2 Department of Respiratory Medicine, Kawasaki Medical School, 577, Matsushima, Kurashiki, Okayama 701-0192 Japan; 3 Department of Thoracic Surgery, Graduate School of Medicine, Nagoya University, Nagoya, Japan; 4 Organ Transplant Center, Okayama University Hospital, Okayama, Japan; 5 Department of Thoracic Surgery, Institute of Development, Aging and Cancer, Tohoku University, Sendai, Japan; 6 Division of Organ Transplantation, Tohoku University Hospital, Sendai, Japan; 7 Department of Respiratory Care and Sleep Control Medicine, Graduate School of Medicine, Kyoto University, Kyoto, Japan

**Keywords:** Lung transplantation, Waitlist mortality, Health-related quality of life, Modified Medical Research Council dyspnea scale, St. George’s Respiratory Questionnaire

## Abstract

**Background:**

Waitlist mortality due to donor shortage for lung transplantation is a serious problem worldwide. Currently, the selection of recipients in Japan is mainly based on the registration order. Hence, scientific evidence for risk stratification regarding waitlist mortality is urgently needed. We hypothesized that patient-reported dyspnea and health would predict mortality in patients waitlisted for lung transplantation.

**Methods:**

We analyzed factors related to waitlist mortality using data of 203 patients who were registered as candidates for lung transplantation from deceased donors. Dyspnea was evaluated using the modified Medical Research Council (mMRC) dyspnea scale, and the health status was determined with St. George’s Respiratory Questionnaire (SGRQ).

**Results:**

Among 197 patients who met the inclusion criteria, the main underlying disease was interstitial lung disease (99 patients). During the median follow-up period of 572 days, 72 patients died and 96 received lung transplantation (69 from deceased donors). Univariable competing risk analyses revealed that both mMRC dyspnea and SGRQ Total score were significantly associated with waitlist mortality (*p* = 0.003 and *p* < 0.001, respectively) as well as age, interstitial lung disease, arterial partial pressure of carbon dioxide, and forced vital capacity. Multivariable competing risk analyses revealed that the mMRC and SGRQ score were associated with waitlist mortality in addition to age and interstitial lung disease.

**Conclusions:**

Both mMRC dyspnea and SGRQ score were significantly associated with waitlist mortality, in addition to other clinical variables such as patients’ background, underlying disease, and pulmonary function. Patient-reported dyspnea and health may be measured through multi-dimensional analysis (including subjective perceptions) and for risk stratification regarding waitlist mortality.

**Supplementary Information:**

The online version contains supplementary material available at 10.1186/s12931-021-01715-x.

## Background

Lung transplantation has been recognized as an effective treatment for patients with various end-stage pulmonary diseases; however, shortage of lung donors is a major challenge worldwide. Due to the imbalance between demand for donor organs and supply, waitlist mortality remains high [1]. In Japan, the average waiting time for transplantation is > 800 days, resulting in a considerable number of deaths while on the waitlist [2]. Therefore, measures to reduce waitlist mortality are urgently warranted. For this purpose, appropriate risk stratification of patients waitlisted for lung transplantation is necessary.

In the U.S., the lung allocation score (LAS) system was implemented in 2005 to reduce the waitlist time and mortality, as well as to improve the likelihood of 1-year post-transplantation survival [3]. The LAS system has been adopted by Eurotransplant [4] and will be possibly introduced in various countries including Japan. This system is primarily based on multi-dimensional analyses; it is weighted based on underlying diseases and calculated mostly through physiological measures [5].

Dyspnea and health-related quality of life (HRQL) are the main targets for improvement after lung transplantation, in addition to survival. Nevertheless, the ranking of these subjective measures in the assessment of patients waitlisted for lung transplantation remains unclear. These patient-reported measures are not included in the factors contributing to the LAS; also, the LAS does not reflect them [6]. This indicates that they should be assessed separately from the LAS.

Assessment of dyspnea and HRQL using questionnaires in patients with respiratory diseases is mainly based on preceding experience and information obtained from studies of chronic airway diseases, such as asthma and chronic obstructive pulmonary disease (COPD). Dyspnea is the main symptom in these patients and a major contributor to poor health. Assessment of dyspnea [7] and health status [8] provides useful prognostic information regarding mortality in patients with COPD, thereby contributing to the development of subsequent multi-dimensional analysis not solely based on pulmonary function [9, 10].

Therefore, we hypothesized that patient-reported dyspnea and HRQL would predict waitlist mortality in patients waitlisted for lung transplantation in Japan, even though their underlying diseases are heterogeneous. This may provide useful information for better stratifying patients at risk of mortality during limited waiting time. We subsequently assessed the relationship between baseline variables, including patient-reported outcomes at registration of lung transplantation and waitlist mortality.

## Methods

### Patients

A total of 203 patients who were registered as candidates for lung transplantation from deceased donors were consecutively recruited from three facilities (Kyoto University Hospital, Okayama University Hospital, and Tohoku University Hospital) in Japan between March 2009 and June 2015 [11]. The inclusion criteria were: (1) new registration in the Japan Organ Transplant Network; (2) age > 18 years; and (3) absence of uncontrolled comorbidities, such as malignant, cardiac, and cerebrovascular diseases. The exclusion criteria were: (1) necessity of heart–lung transplantation; and (2) refusal to participate in the study. Patients’ background, pulmonary function, arterial blood gas, and patient-reported measurements of dyspnea, HRQL, and psychological status were assessed at study entry. Comorbidity was objectively assessed using the Charlson comorbidity index [12]. Patients underwent spirometry to measure their forced vital capacity (FVC) and forced expiratory volume in the first second (FEV_1_). Predicted values were based on the recommendation by the Japanese Respiratory Society guidelines. The study protocol (E554) was approved by the Ethics Committees of all facilities participating in this research. Written informed consent was provided by all patients.

### Patient-reported outcomes

Dyspnea was evaluated using the Japanese version of the modified Medical Research Council (mMRC) dyspnea scale [8, 9]. This is a 5-point scale (0–4) based on the degrees of various physical activities that precipitate dyspnea; higher scores indicate a worse status.

Heath status or HRQL was assessed using the Japanese version of St. George’s Respiratory Questionnaire (SGRQ) [8, 13]. The SGRQ was originally developed for patients with chronic airflow limitations, such as asthma or COPD. However, it has been validated for use in other respiratory diseases, including pulmonary fibrosis, bronchiectasis, lymphangioleiomyomatosis, pulmonary hypertension, and bronchiolitis obliterans. Therefore, it is the only frequently employed respiratory-specific HRQL instrument in lung transplantation [14]. It consists of 50 items divided into three components: Symptoms, Activities, and Impacts. The Total SGRQ score was calculated, with scores ranging from 0 (best) to 100 (worst).

Psychological status was evaluated using the Japanese version of the Hospital Anxiety and Depression Scale (HADS) [11, 15], which consists of 14 items: seven for anxiety and seven for depression. Each item is scored from 0 to 3, where a score of 3 represents a state corresponding to the worst anxiety or depression. The sum of these items produces two subscale scores, each ranging from 0 to 21.

### LAS

The LAS of each patient was calculated using the LAS calculator on the Organ Procurement and Transplantation Network website (http://optn.transplant.hrsa.gov/resources/allocation-calculators/las-calculator/) in September 2020. In this study, the LAS was calculated using the data at the time of first study registration.

### Statistical analysis

Results are presented as the median with interquartile range, unless otherwise stated. The duration from entry to the time of waitlist death (event), transplantation date including transplantations both from deceased and living donors (competing risk), or confirmation of survival without transplantation up to 5 years were recorded. Patients who withdrew from the study or remained on the waitlist were censored. We truncated the time-varying curves when < 10 subjects were at risk.

Univariable and multivariable competing risk regressions with the Fine–Gray model [16] were performed to investigate the relationship between clinical measurements and waitlist mortality based on previous analyses [7, 8, 17]. FVC (%predicted), interstitial lung disease (ILD), mMRC dyspnea, SGRQ, and HADS were included in the multivariable analyses based on clinical experience even if they were not significant variables in the univariable analyses. Other than these variables, those with *p*-values < 0.05 were included in the multivariable analyses. Clinical measurements were defined as continuous variables with the exception of sex, smoking status, use of home oxygen therapy, and kinds of primary underlying diseases (ILD or other). The results of the regression analysis were presented as estimated hazard ratios (HRs) with corresponding 95% confidence intervals (CIs). Competing risk regression with the Fine–Gray model was used to evaluate waitlist mortality in the groups with lower versus higher mMRC dyspnea, lower versus higher SGRQ score, and lower versus higher LAS based on the median score. The *p*-values < 0.05 denoted statistically significant differences. Statistical analyses were performed using the statistical software EZR (Saitama Medical Center, Jichi Medical University, Saitama, Japan).

## Results

### Patients

Among the 203 patients enrolled, six patients were excluded (four were lost to follow-up and two were transferred to other hospitals immediately after registration). Therefore, 197 patients (102 males) who met the inclusion criteria were analyzed; their baseline characteristics are presented in Table 1. The study population had a median age of 47 (38–54) years and low body mass index of 19.0 (16.7–21.7) kg/m^2^. The primary underlying diseases were ILD (99 patients, 50.3%), bronchiolitis obliterans (21 patients, 10.7%), pulmonary hypertension (15 patients, 7.6%), lymphangioleiomyomatosis (15 patients, 7.6%), COPD (12 patients, 6.1%), bronchiectasis (11 patients, 5.6%), and lung injury after hematopoietic stem cell transplantation (10 patients, 5.1%). Long-term oxygen therapy and noninvasive ventilation were used for 174 and 11 patients, respectively. FVC and FEV_1_ values were low at 46.4 (36.1–61.6) %predicted and 40.8 (24.5–55.8) %predicted, respectively.

**Table 1:** Baseline characteristics of 197 patients waiting for lung transplantation

Characteristics	Median or number	IQR
Sex, male/female, n	102/95	
Age, years	47	38–54
BMI, kg/m^2^	19.0	16.7–21.7
Smokers, number	94 (47.7%)	
Charlson comorbidity index	0	0–1
PaO_2_, mmHg	74.5	62.0–88.9
PaCO_2_, mmHg	45.1	40.1–51.5
FVC, L	1.7	1.2–2.3
FVC, %predicted	46.4	36.1–61.6
FEV_1_, L	1.2	0.7–1.7
FEV_1_, %predicted	40.8	24.5–55.8
6-min walk distance, m	300	208–389
mMRC dyspnea (0–4)	3	2–4
SGRQ symptoms (0–100)	69.6	56.2–80.7
SGRQ activities (0–100)	86.4	77.2–92.5
SGRQ impacts (0–100)	57.5	43.3–70.1
SGRQ total (0–100)	67.3	56.5–76.7
HADS anxiety (0–21)	5	2–8
HADS depression (0–21)	6	3–9

Numbers in parentheses indicate the theoretical score range

There were some unavailable data: PaO_2_ and PaCO_2_ were measured in 195 patients, FVC and FEV_1_ were measured in 191 patients, and 6-min walk distance was measured in 179 patients

*IQR* interquartile range, *BMI* body mass index, *PaO*_*2*_ arterial partial pressure of oxygen, *PaCO*_*2*_ arterial partial pressure of carbon dioxide, *FVC* forced vital capacity, *FEV*_*1*_ forced expiratory volume in the first second, *mMRC* modified Medical Research Council, *SGRQ* St. George’s Respiratory Questionnaire, *HADS* Hospital Anxiety and Depression Scale

### Prospective survival study

During the median follow-up period of 572 days (range: 1–1987 days), 96 patients (48.7%) received lung transplantation. Among them, 27 patients underwent living-donor lung lobar transplantation because they could not wait for a deceased donor. Regarding the 69 patients who underwent lung transplantation from a deceased donor, the median time from registration to lung transplantation was 752 days (range: 38–1979 days). Of note, 72 patients on the waitlist (36.5%) died: 49 had ILD, and 23 had other respiratory diseases. With regard to the cause of death, 70 patients (97.2%) died due to worsening of the original respiratory diseases; the remaining two patients died due to pneumonia and pulmonary hemorrhage (one patient each).

**Fig. 1:**
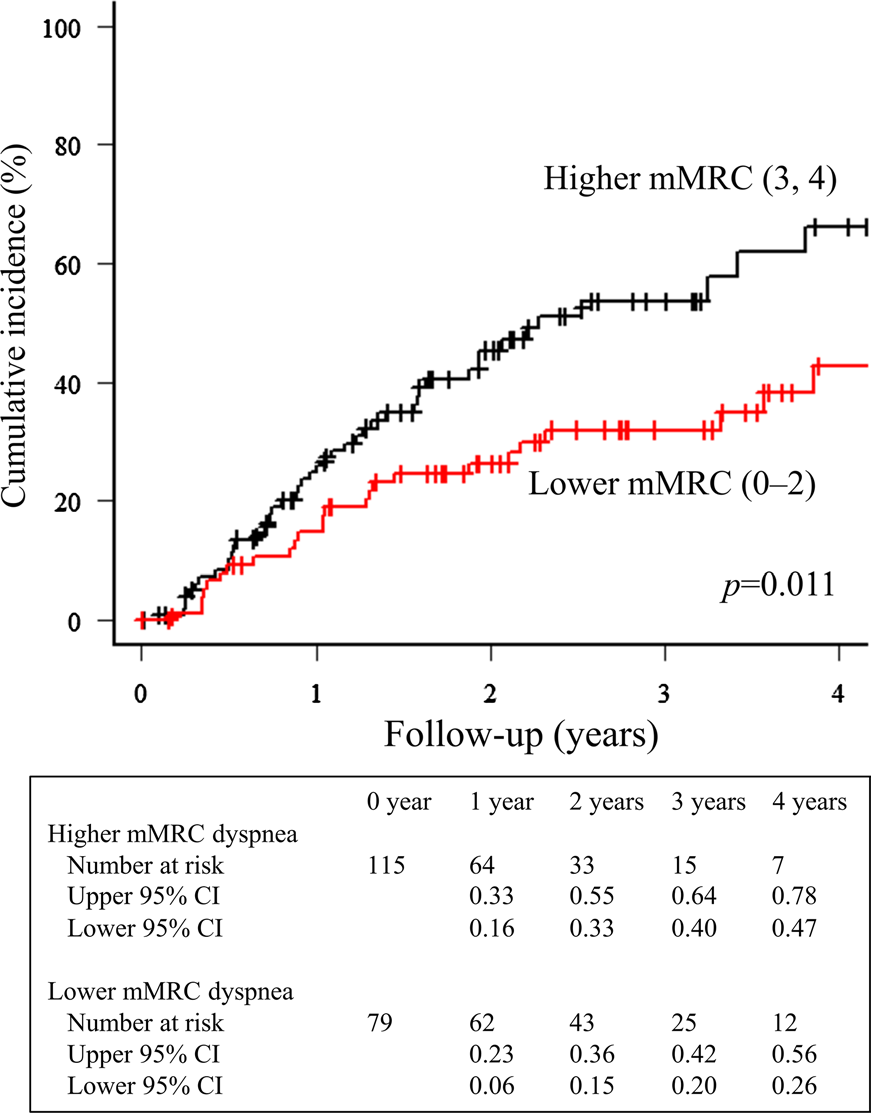
Cumulative incidence on the waiting list comparing groups with higher and lower mMRC dyspnea based on the median score. The group with higher mMRC scores (mMRC = 3 or 4) showed a significantly higher waitlist mortality rate than that with lower mMRC scores (mMRC = 0–2) (*p* = 0.011). *CI* confidence interval, *mMRC* modified Medical Research Council

**Fig. 2:**
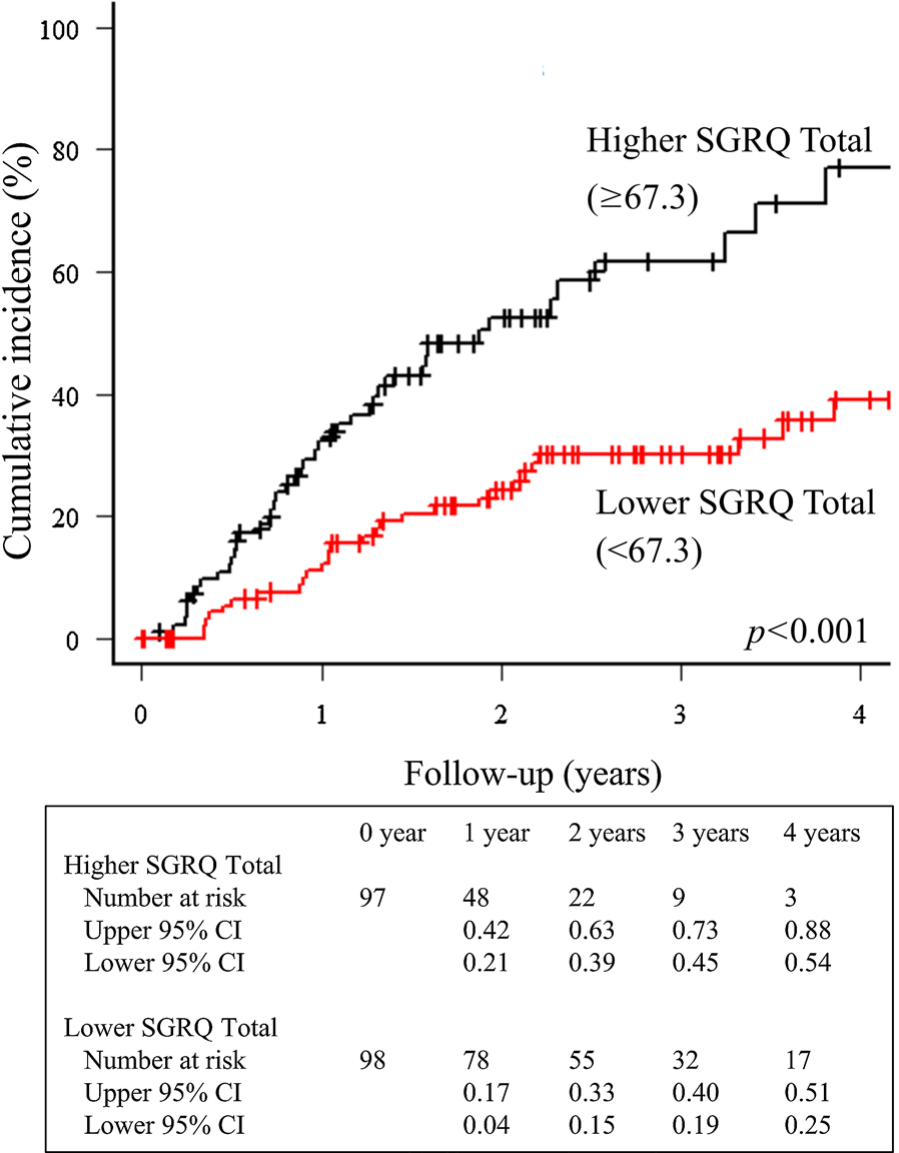
Cumulative incidence on the waiting list comparing groups with higher and lower SGRQ Total score based on the median score. The group with higher SGRQ Total scores showed a significantly higher waitlist mortality rate than that with lower SGRQ Total scores (*p* < 0.001). *CI* confidence interval, *SGRQ* St. George’s Respiratory Questionnaire

Univariable competing risk regressions with the Fine–Gray model were performed to investigate the relationships between clinical measurements and waitlist mortality (Table 2). Higher age, ILD, higher arterial partial pressure of carbon dioxide (PaCO_2_), and lower FVC were significantly related to waitlist mortality (*p* < 0.05). Regarding patient-reported measures, mMRC dyspnea was significantly related to waitlist mortality (HR = 1.46, 95% CI 1.13–1.87, *p* = 0.003). For patients classified according to the median mMRC (3), cumulative incidence curves between the lower and higher mMRC score groups (score 0–2, n = 79; score 3 or 4, n = 115; missing, n = 3) are presented (*p* = 0.011) (Fig. 1). The HRQL, Symptoms, Activities, Impacts, and Total scores of the SGRQ were strongly associated with waitlist mortality (HR = 1.02, 95% CI 1.01–1.04, *p* = 0.0014; HR = 1.03, 95% CI 1.01–1.05, *p* = 0.002; HR = 1.03, 95% CI 1.02–1.04, *p* < 0.001; and HR = 1.04, 95% CI 1.02–1.05, *p* < 0.001, respectively). For patients classified according to the median SGRQ Total score (67.3), cumulative incidence curves between the higher and lower SGRQ Total score groups (n = 97 and n = 98, respectively; missing, n = 2) are presented (*p* < 0.001) (Fig. 2). HADS anxiety and depression were also weakly but significantly related to waitlist mortality (HR = 1.07, 95% CI 1.02–1.13, *p* = 0.013; and HR = 1.05, 95% CI 1.01–1.10, *p* = 0.024, respectively). Other variables, such as sex, body mass index, smoking history, comorbidities, long-term oxygen therapy, FEV_1_, and 6-min walk distance were not significantly related to waitlist mortality (*p* > 0.05).

**Table 2:** Univariable competing risk analysis with the Fine–Gray model in 197 patients waiting for lung transplantation

Characteristics	HR	95% CI	*p-*value
Sex, male	1.08	0.68–1.71	0.76
Age, years	1.04	1.02–1.06	< 0.001
BMI, kg/m^2^	1.00	0.94–1.05	0.90
Smoking history	1.01	0.64–1.62	0.96
Charlson comorbidity index	1.01	0.75–1.37	0.96
Interstitial lung disease	2.75	1.70–4.44	< 0.001
Long-term oxygen therapy	0.80	0.43–1.52	0.50
PaO_2_, mmHg	1.01	1.00–1.02	0.14
PaCO_2_, mmHg	1.02	1.00–1.04	0.041
FVC, L	0.58	0.43–0.80	< 0.001
FVC, %predicted	0.98	0.97–0.99	< 0.001
FEV_1_, L	0.92	0.68–1.25	0.61
FEV_1_, %predicted	1.00	0.99–1.01	0.89
6-min walk distance, m	1.00	1.00–1.00	0.55
mMRC dyspnea	1.46	1.13–1.87	0.003
SGRQ symptoms	1.02	1.01–1.04	0.0014
SGRQ activities	1.03	1.01–1.05	0.002
SGRQ impacts	1.03	1.02–1.04	< 0.001
SGRQ total	1.04	1.02–1.05	< 0.001
HADS anxiety	1.07	1.02–1.13	0.013
HADS depression	1.05	1.01–1.10	0.024

*HR* hazard ratio, *CI* confidence interval, *BMI* body mass index, *PaO*_*2*_ arterial partial pressure of oxygen, *PaCO*_*2*_ arterial partial pressure of carbon dioxide, *FVC* forced vital capacity, *FEV*_*1*_ forced expiratory volume in the first second, *mMRC* modified Medical Research Council, *SGRQ* St. George’s Respiratory Questionnaire, *HADS* Hospital Anxiety and Depression Scale

Multivariable competing risk regression with the Fine–Gray model was performed using age, ILD, PaCO_2_, FVC (%predicted), and mMRC (Model I: dyspnea) or SGRQ Total score (Model II: HRQL) or HADS (Model III: psychological status) as explanatory variables; all were significant factors in the univariable analyses (Table 3). We divided them into these three groups because the SGRQ Total score includes the evaluation of dyspnea and psychological status. In Model I, age, ILD, FVC, and mMRC dyspnea were significantly related to waitlist mortality (HR = 1.04, *p* = 0.007; HR = 2.41, *p* = 0.002; HR = 0.98, *p* = 0.016; and HR = 1.36, *p* = 0.037, respectively). In Model II, age, ILD, and SGRQ Total score were significantly related to waitlist mortality (HR = 1.03, *p* = 0.010; HR = 2.24, *p* = 0.003; and HR = 1.03, *p* = 0.0014, respectively). In Model III, age, ILD and FVC were significantly related to waitlist mortality, whereas HADS was not. We also analyzed the data excluding patients who underwent living-donor lobar lung transplantation after registration in this study. We found that the SGRQ Total score and mMRC dyspnea remained significantly related to waitlist mortality after multivariable analysis (Additional file 1: Table S1).

**Table 3:** Multivariable competing risk analysis with the Fine–Gray model to analyze the relationship between patient-reported outcomes and mortality in patients waiting for lung transplantation

	Model I (dyspnea)			Model II (HRQL)			Model III (psychological status)		
	HR	95% CI	*p*-value	HR	95% CI	*p*-value	HR	95% CI	*p-*value
Age, years	1.04	1.01–1.06	0.007	1.03	1.01–1.06	0.010	1.04	1.01–1.06	0.002
Interstitial lung disease	2.41	1.38–4.21	0.002	2.24	1.32–3.78	0.003	2.10	1.26–3.51	0.005
PaCO_2_, mmHg	1.00	0.97–1.03	0.950	1.00	0.97–1.04	0.840	1.00	0.96–1.03	0.830
FVC, %predicted	0.98	0.96–1.00	0.016	0.99	0.97–1.01	0.150	0.98	0.96–0.99	0.002
mMRC dyspnea	1.36	1.02–1.81	0.037						
SGRQ total				1.03	1.01–1.05	0.0014			
HADS anxiety							1.05	0.95–1.15	0.330
HADS depression							1.00	0.93–1.07	0.970

*HRQL* health-related quality of life, *HR* hazard ratio, *CI* confidence interval, *PaCO*_*2*_ arterial partial pressure of carbon dioxide, *FVC* forced vital capacity, *mMRC* modified Medical Research Council, *SGRQ* St. George’s Respiratory Questionnaire, *HADS* Hospital Anxiety and Depression Scale

Finally, we analyzed data regarding the LAS. The values ranged from 32.6 to 71.2 (median score: 40.1). Univariable competing risk analysis revealed that the LAS was significantly related to waitlist mortality (HR = 1.04; 95% CI 1.01–1.07, *p* = 0.004). Cumulative incidence curves for the groups with lower versus higher LAS are presented (n = 87 and n = 86, respectively) (*p* = 0.025) (Additional file 2: Figure S1). We subsequently analyzed whether the significant relationship of the LAS with waitlist mortality was independent of patient-reported measures. Therefore, multivariable competing risk analysis was performed to investigate the relationships with waitlist mortality between the LAS and (a) Model I: mMRC score (dyspnea), (b) Model II: SGRQ Total (HRQL), and (c) Model III: HADS anxiety and depression (psychological status). In Model III, only the LAS was significant (*p* = 0.012); in Model I, both the LAS and the mMRC were significantly related to waitlist mortality (*p* = 0.039 and 0.038, respectively); and in Model II, the SGRQ was significantly related to waitlist mortality (*p* < 0.001), whereas the LAS was not (*p* = 0.11) (Additional file 3: Table S2).

## Discussion

In the present study, we analyzed factors related to waitlist mortality in patients waiting for lung transplantation in Japan, focusing on patient-reported outcomes. The most important novel finding was that mMRC dyspnea and HRQL assessed using the SGRQ were significantly associated with waitlist mortality, in addition to other clinical variables such as age, underlying disease (ILD or non-ILD), and pulmonary function.

It is noteworthy that the patient-reported dyspnea and health were significantly associated with waitlist mortality in patients waiting for lung transplantation in the multivariable models. Some aspects of these sensory symptoms and experience can be objectively measured using questionnaires, enabling us to differentiate between patients with better or worse status (discriminative property) and assess their degree of change, e.g., in response to medical intervention (evaluative property) [18]. Subsequent studies reported that this approach can predict future outcomes, such as mortality in patients with COPD (predictive property) [7, 8]. Regarding idiopathic pulmonary fibrosis (IPF), the most prevalent original disease in the present study, mMRC dyspnea [19] and SGRQ [20, 21] also predicted mortality. Notably, well-established multi-dimensional severity scoring for IPF, such as the composite physiologic index [22] or GAP (gender, age and physiology) [23], did not include these measures.

However, we demonstrate for the first time that the predictive properties of both the mMRC and SGRQ score were advantageously maintained in this heterogeneous group of patients with various advanced lung disorders and targeted for lung transplantation. This information will help us to more accurately stratify patients at risk for waitlist mortality and better cope with this serious problem. These patients often have severe disease limiting their ability to perform important physiological tests, such as diffusing capacity or 6-min walk. Therefore, the use of appropriate questionnaires simplifies the assessment of dyspnea and HRQL in patients. Unlike pulmonary function, dyspnea and health can reflect systemic as well as local comprehensive effects of the disease on patients [7, 8].

Unfortunately, waitlist mortality remains a significant problem [1]. According to U.S. reports [1, 24], approximately 20% of waitlisted patients died while on the lung transplantation waitlist or are removed because they become ineligible for transplantation due to severe disease. Nevertheless, this mortality rate appears to be markedly lower than that observed in the present study (36.5%). Long waiting time for lung transplantation is a serious problem in Japan and numerous other countries; hence, it is important to reduce waitlist mortality as much as possible.

Currently, there are nine certified lung transplantation centers in Japan, including Kyoto University. All potential recipients are evaluated by the nationwide Central Lung Transplant Evaluation Committee following acceptance by the Lung Transplant Evaluation Committee at each lung transplantation center [2]. Subsequently, the accepted candidates are registered on the waitlist of the Japan Organ Transplant Network Center. The selection of lung transplantation recipients in Japan is currently based on a “first come, first served” system, in addition to blood type compatibility, organ size, and other factors. The LAS system in the U.S. appears to function effectively. Numerous countries, including Japan, are attempting to develop such systems depending on their specific circumstances for lung transplantation. Given that the development of such systems may require considerable time, the present study was conducted to yield some scientific evidence with regard to risk stratification for organ allocation.

We calculated the LAS as a reference value, despite some missing values. Interestingly, the mMRC and SGRQ score were significantly related to mortality independently of the LAS. Thus, these subjective measures may add values to the existing lung allocation system in terms of patient risk stratification and patient perception. However, further prospective studies are warranted to examine this hypothesis.

ILD was significantly associated with waitlist mortality in different multivariable models. Among the 99 patients with ILD enrolled in this study, 49 patients (49.5%) on the waitlist died. This is consistent with historical evidence [4] showing that patients with IPF have a higher waitlist mortality rate than those with other common indications for transplantation (67% versus 18%, respectively). In the U.S., the primary indications are fibrotic lung disorders, currently accounting for > 57% of cases [1]. Similarly, in the present Japanese study, ILD accounted for 50.3% of the 197 analyzed patients. Thus, ILD occupies a significant place in lung transplantation regarding incidence and waitlist mortality, which should be solved in the future.

Additionally, higher PaCO_2_ was an important factor for mortality. A previous retrospective study of patients with IPF in a single center yielded similar results [25]. The present prospective study corroborated these findings among heterogenous patients waiting for transplantation, not limited to IPF. In the future, we wish to investigate the potential usefulness of PaCO_2_ as a new biomarker for risk stratification.

Some limitations of the present study should be mentioned. Firstly, the present study was conducted in Japan where the LAS system is not used and the waitlist time is long. In Japan, allocation is solely based on the waiting time. In contrast, in the Eurotransplant region and the U.S., an allocation system is in place, stratifying patients based on medical urgency and anticipated 1-year survival. In addition, the underlying diseases of enrolled patients may differ between countries. Therefore, the present results may be specific to Japan. Moreover, because of the small number of patients, we were unable to perform an analysis based on each underlying disease. Our findings were only adjusted depending on the presence or absence of interstitial pneumonia through multivariable analysis. Hence, further study is warranted to determine the generalization of these findings. Secondly, we used mMRC and SGRQ score as measures of dyspnea and HRQL, respectively, both of which are well-established questionnaires in the respiratory field. However, as they are not specific to patients with severe diseases, the median and whole scores tended to skew toward worse outcomes. Lung transplant-specific instruments, such as the recent Lung Transplant Quality of Life (LT-QOL) [26], may generate better predictive features. Unfortunately, we did not have detailed data at the time of transplantation. This approach could provide insight into the association of mMRC and SGRQ score with the course of disease, in addition to mortality.

## Conclusions

In this study, patient-reported dyspnea and health were significantly associated with waitlist mortality in patients waiting for lung transplantation, in addition to other clinical variables such as patients’ age, underlying disease, and physiological measures. Patient-reported dyspnea and health may be measured through multi-dimensional analysis, including subjective perceptions. Following the development of a new allocation system in Japan, these parameters can be used for risk stratification regarding waitlist mortality.

## Supplementary Information

**Additional file 1: Table S1.** Multivariable Fine–Gray proportional hazards analysis of the relationship between patient-reported outcomes and mortality in waitlisted patients, excluding those who underwent living-donor lung lobar transplantation after registration.

**Additional file 2: Figure S1.** Cumulative incidence on the waiting list comparing groups with higher and lower LAS scores based on the median score. CI, confidence interval; LAS, lung allocation score

**Additional file 3: Table S2.** Multivariable Fine–Gray proportional hazards analysis of the relationship of LAS and patient-reported outcomes with mortality in patients waiting for lung transplantation.

## Data Availability

The datasets used and/or analyzed during the present study are available from the corresponding author upon reasonable request.
