# Supplementary material for: Patient-reported dyspnea and health predict waitlist mortality in patients waiting for lung transplantation in Japan
Source: Respir Res. 2021 Apr 21;22:116. doi: 10.1186/s12931-021-01715-x (PMC8061007; doi:10.1186/s12931-021-01715-x)
Supplement: Supplementary file 1 — Additional file 1: Table S1. Multivariable Fine–Gray proportional hazards analysis of the relationship between patient-reported outcomes and mortality in waitlisted patients, excluding those who underwent living-donor lung lobar transplantation after registration. [file 12931_2021_1715_MOESM1_ESM.docx]

**Table S1. Multivariable Fine–Gray proportional hazards analysis of the relationship between patient-reported outcomes and mortality in waitlisted patients, excluding those who underwent living-donor lung lobar transplantation after registration**

|  | Model I (dyspnea) | | | Model II (HRQL) | | | Model III (psychological status) | | |
| --- | --- | --- | --- | --- | --- | --- | --- | --- | --- |
|  | HR | 95% CI | *p-*value | HR | 95% CI | *p-*value | HR | 95% CI | *p-*value |
| Age, years | 1.04 | 1.01–1.07 | 0.007 | 1.04 | 1.01–1.06 | 0.010 | 1.04 | 1.01–1.07 | 0.002 |
| IP | 2.41 | 1.38–4.19 | 0.002 | 2.26 | 1.33–3.82 | 0.002 | 2.09 | 1.25–3.47 | 0.005 |
| PaCO_2_, mmHg | 1.00 | 0.97–1.04 | 0.880 | 1.00 | 0.97–1.04 | 0.800 | 1.00 | 0.96–1.03 | 0.900 |
| FVC, %predicted | 0.98 | 0.97–1.00 | 0.017 | 0.99 | 0.97–1.01 | 0.180 | 0.98 | 0.96–0.99 | 0.002 |
| mMRC dyspnea | 1.36 | 1.01–1.81 | 0.040 |  |  |  |  |  |  |
| SGRQ Total |  |  |  | 1.03 | 1.01–1.05 | 0.001 |  |  |  |
| HADS anxiety |  |  |  |  |  |  | 1.05 | 0.96–1.16 | 0.300 |
| HADS depression |  |  |  |  |  |  | 1.00 | 0.93–1.07 | 0.940 |

HR, hazard ratio; CI, confidence interval; PaCO_2_, arterial partial pressure of carbon dioxide; FVC, forced vital capacity; HRQL, health-related quality of life; mMRC, modified Medical Research Council; SGRQ, St. George’s Respiratory Questionnaire; HADS, Hospital Anxiety and Depression Scale.
