# Supplementary figures and images for: Patient-reported dyspnea and health predict waitlist mortality in patients waiting for lung transplantation in Japan
Source: Respir Res. 2021 Apr 21;22:116. doi: 10.1186/s12931-021-01715-x (PMC8061007; doi:10.1186/s12931-021-01715-x)

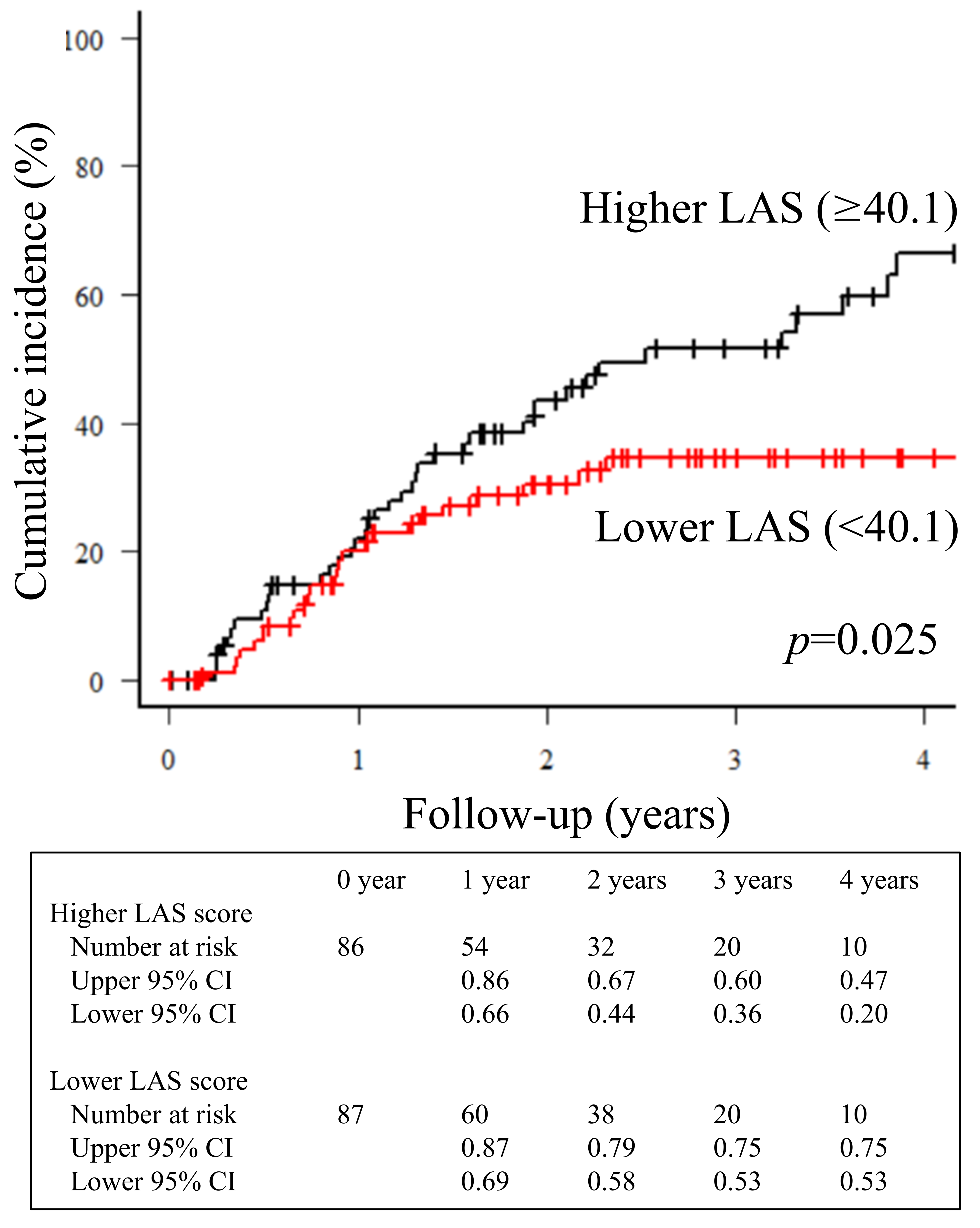

Supplement: Supplementary file 2 — Additional file 2: Figure S1. Cumulative incidence on the waiting list comparing groups with higher and lower LAS scores based on the median score. CI, confidence interval; LAS, lung allocation score [file 12931_2021_1715_MOESM2_ESM.tif]
