# Supplementary material for: Patient-reported dyspnea and health predict waitlist mortality in patients waiting for lung transplantation in Japan
Source: Respir Res. 2021 Apr 21;22:116. doi: 10.1186/s12931-021-01715-x (PMC8061007; doi:10.1186/s12931-021-01715-x)
Supplement: Supplementary file 3 — Additional file 3: Table S2. Multivariable Fine–Gray proportional hazards analysis of the relationship of LAS and patient-reported outcomes with mortality in patients waiting for lung transplantation. [file 12931_2021_1715_MOESM3_ESM.docx]

**Table S2. Multivariable Fine–Gray proportional hazards analysis of the relationship of LAS and patient-reported outcomes with mortality in patients waiting for lung transplantation**

|  | Model I (dyspnea) | | | Model II (HRQL) | | | Model III (psychological status) | | |
| --- | --- | --- | --- | --- | --- | --- | --- | --- | --- |
|  | HR | 95% CI | *p*-value | HR | 95% CI | *p*-value | HR | 95% CI | *p*-value |
| LAS | 1.03 | 1.00–1.06 | 0.039 | 1.03 | 0.99–1.06 | 0.110 | 1.04 | 1.01–1.07 | 0.012 |
| mMRC dyspnea | 1.32 | 1.02–1.72 | 0.038 |  |  |  |  |  |  |
| SGRQ Total |  |  |  | 1.03 | 1.02–1.05 | <0.001 |  |  |  |
| HADS anxiety |  |  |  |  |  |  | 1.07 | 0.98–1.18 | 0.110 |
| HADS depression |  |  |  |  |  |  | 0.99 | 0.92–1.06 | 0.740 |

HR, hazard ratio; CI, confidence interval; LAS, lung allocation score; HRQL, health-related quality of life; mMRC, modified Medical Research Council; SGRQ, St. George’s Respiratory Questionnaire; HADS, Hospital Anxiety and Depression Scale.
